# Correction to: Male mate choice in a sexually cannibalistic species: male escapes from hungry females in the praying mantid *Tenodera angustipennis*

**DOI:** 10.1007/s10164-017-0534-8

**Published:** 2017-12-01

**Authors:** Mika Kadoi, Kotaro Morimoto, Yasuoki Takami

**Affiliations:** 1 Faculty of Human Development, Kobe University, Tsurukabuto 3-11, Nada, Kobe 657-8501 Japan; 2 Graduate School of Human Development and Environment, Kobe University, Tsurukabuto 3-11, Nada, Kobe 657-8501 Japan

## Correction to: J Ethol (2017) 35:177–185 10.1007/s10164-017-0506-z

The article, “Male mate choice in a sexually cannibalistic species: male escapes from hungry females in the praying mantid *Tenodera angustipennis*”, written by Mika Kadoi, Kotaro Morimoto and Yasuoki Takami, was originally published Online First without open access. After publication in volume 35, issue 2, page 177–185 the author decided to opt for Open Choice and to make the article an open access publication. Therefore, the copyright of the article has been changed to © The Author(s) 2018 and the article is forthwith distributed under the terms of the Creative Commons Attribution 4.0 International License (http://creativecommons.org/licenses/by/4.0/), which permits use, duplication, adaptation, distribution and reproduction in any medium or format, as long as you give appropriate credit to the original author(s) and the source, provide a link to the Creative Commons license, and indicate if changes were made.

